# Perceived environmental impact of meat alternatives: Effects of a sustainability label and product type

**DOI:** 10.1016/j.crfs.2025.101254

**Published:** 2025-11-27

**Authors:** Dacinia Crina Petrescu, Ruxandra Malina Petrescu-Mag, Melis Aras, Marius Bota, Alexandru Sut

**Affiliations:** a Faculty of Business, Babes-Bolyai University, 7 Horea Street, Cluj-Napoca, 400174, Romania; b Department of Economy and Rural Development, Faculty of Gembloux Agro-Bio Tech, University of Liège, Passage des Déportés, 2, Gembloux, 5030, Belgium; c Faculty of Environmental Science and Engineering, Babes-Bolyai University, 30 Fantanele Street, Cluj-Napoca, 400294, Romania; d Doctoral School “International Relations and Security Studies”, Babes-Bolyai University, 1 Mihail Kogalniceanu Street, Cluj-Napoca, 400084, Romania; e The Law and Social Change (DCS) Laboratory - UMR CNRS 6297, Faculty of Law, Nantes University, Chemin de la Censive du Tertre BP 81307, 44 313, Nantes, Cedex 3, France

**Keywords:** Meat alternative, Insect, Cultured meat, Plant-based, Climate change, Animal welfare, Protein

## Abstract

Growing environmental awareness and interest in sustainable, nutritious diets fueled the rapid expansion of meat alternatives. However, consumer acceptance remains uneven, and responses to sustainability labels are ambiguous, particularly for novel protein sources. This study investigates how a sustainability label and product type influence consumers’ perceptions of the environmental impact of meat alternatives and meat products. A 2 (label: sustainability label; no label) × 4 (product type: insect-based; cultured meat; plant-based; beef burgers) between-subjects experiment was conducted with 432 Romanian consumers. Perceived environmental impact was assessed across three dimensions – resource consumption, climate change, and animal welfare – using two-way MANOVA. The sustainability label had limited effects: only for insect-based burgers, and it unexpectedly increased perceived climate harm. For all other products, labels did not shift evaluations. Beef burgers were constantly perceived as the most damaging. An interaction effect between label and product type emerged only for perceptions of climate change. This research presents the first integrated analysis of label × product type effects across multiple environmental dimensions, comparing meat alternatives and meat products. It highlights that labels may not always enhance positive perceptions and can, for less familiar products, amplify negative judgments. Marketers of novel proteins should align sustainability messaging with product familiarity, while beef producers may benefit from highlighting verifiable environmental improvements. Policymakers may consider developing dimension-specific labeling standards and implementing consumer education initiatives to enhance the effectiveness of labels. Enhancing public understanding of sustainability claims can help align consumer perceptions with scientific evidence, supporting responsible marketing and sustainable dietary choices.

## Highlights

• The effects of a sustainability label and product type on perceptions of the environmental impact of a product are analyzed.
• Sustainability labels rarely change perceptions of meat alternatives.
• Sustainability labels increased the perceived climate harm of an insect burger.
• Beef burgers are viewed as the most damaging across all dimensions.
• Label × product type interaction was found only for climate perceptions.

## Graphical abstract

**Figure:**
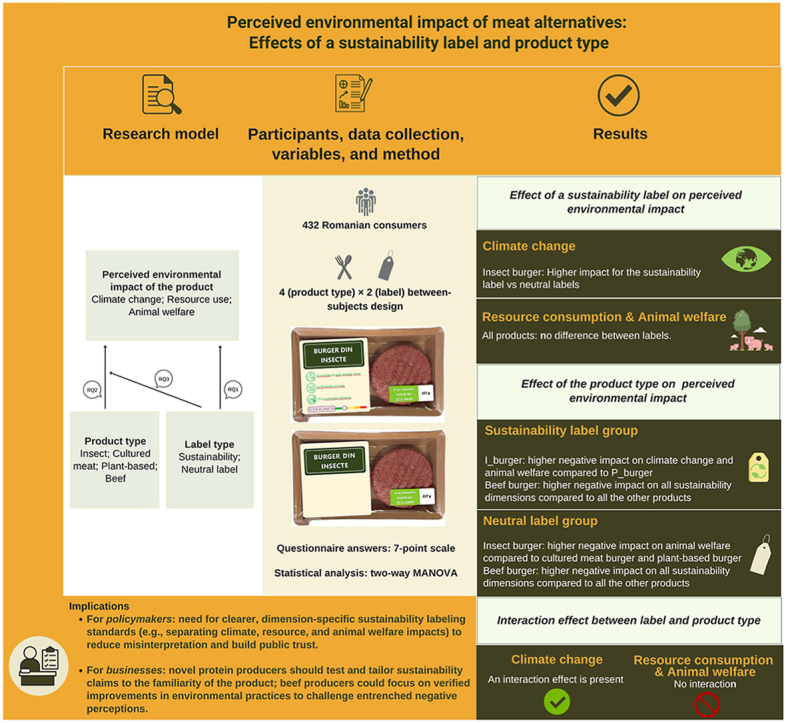

## 1 Introduction

Food systems require transformation to effectively address the interconnected challenges of biodiversity loss, climate change, and public health, but this transformation is challenging (Runhaar, 2025). Interest in meat alternatives, such as cultured meat, insect-based, and plant-based products, has surged in recent years due to their potential to address critical environmental, health, and ethical challenges (Ali and Bharali, 2025; Hartmann and Siegrist, 2020; Tso et al., 2020). This growing attention aligns with the principles of the “One Health” concept, which advocates an integrated, systemic, and unifying approach to human, animal (domestic and wild), and plant health, as well as that of ecosystems, at all levels (Adisasmito et al., 2022).

In this context, meat alternatives are closely tied to the United Nations Sustainable Development Goals (SDGs), particularly SDG 13, which calls for urgent climate action. The global food system occupies approximately 30 % of Earth's land surface and is responsible for roughly 20–35 % of all greenhouse gas emissions (Clark et al., 2020). Within this context, livestock production is a significant driver of emissions and land-use change, supplying approximately 37 % of human dietary protein but utilizing over 80 % of agricultural land (Scherer et al., 2023; Poore and Nemecek, 2018). Alternative protein sources, including plant-based, cultured, and insect-derived proteins, can reduce resource consumption by up to 70 % compared to conventional meat, while providing comparable nutritional value (Nirmal et al., 2025). These efficiency gains translate directly into lower carbon footprints, reduced land degradation, and less freshwater withdrawal, thereby advancing the Paris Agreement targets and SDG 13. Meeting this goal will require transformative shifts in dietary patterns, as current consumption habits continue to threaten biodiversity and climate stability (Clark et al., 2020). Integrating meat alternatives into global diets thus represents a practical lever to realign food production with planetary boundaries, simultaneously promoting responsible consumption (SDG 12) and food security (SDG 2) through sustainable resource use and climate-resilient nutrition.

Building on these sustainability imperatives, emerging innovations such as cultured meat exemplify how technological advances can operationalize the environmental goals outlined by the SDGs. Cultured meat, for instance, is engineered to mimic traditional meat products but claims to have significantly lower greenhouse gas emissions and land use compared to conventional livestock farming (Shen and Chen, 2020). Innovation in cell-based meat supports the EU's Food 2030 goals for a sustainable, climate-smart, and health-benefiting food system (European Commission, 2018). However, much interdisciplinary research and technological advancements are still needed for the large-scale production of cultured meat (da Silva Nunes et al., 2025).

Similarly, insect-based products are gaining attention for their sustainable protein content and lower environmental impact (Ke et al., 2025; Sánchez-Muros et al., 2014; Sogari et al., 2023). Insects require far less water, feed, and space compared to traditional livestock, making them a viable option for environmentally conscious consumers (Van Huis and Oonincx, 2017).

Plant-based products offer a range of alternatives that cater to dietary preferences while reducing reliance on animal husbandry. These products not only support healthier eating habits by promoting the consumption of fruits and vegetables but also contribute to lowering carbon emissions associated with the food life cycle (Afrouzi et al., 2023; González-García et al., 2018; Petrescu-Mag et al., 2024).

Despite the multiple benefits associated with meat alternative sources, debates persist over their nutritional adequacy, environmental claims, and ethical implications. One of these refers to the nutritional and potential health advantages in terms of chronic disease prevention, which remain debated and highly speculative (Santo et al., 2020). Critics note that some plant-based substitutes may not match the protein quality or micronutrient content of the meat, raising concerns about their adequacy as a complete dietary replacement (Harnack et al., 2021). Berggren et al. (2019) suggest that the environmental advantages of using insects as food are unclear due to limited understanding of suitable species, housing, and feeding requirements, as well as the potential for unintended release into the environment. Moreover, controversies surrounding meat alternatives extend to broader societal and ethical considerations. Some critics question the sustainability claims of plant-based foods, highlighting issues such as high processing and transport emissions, as well as land use concerns related to monoculture farming practices for ingredients like soy protein (Zuin et al., 2022). Similarly, Chriki and Hocquette (2020), Chriki et al. (2025), and Post et al. (2020) pinpoint the controversy around the reduction of greenhouse gas emissions from cultured meat, and knowledge gaps in meeting quality standards and expectations. Regarding insects, scientists found evidence of pain experienced by various insects and drew attention to the need to reconsider insect welfare standards to protect the interests of all animals (Birch, 2017; Gibbons et al., 2022). Furthermore, other forms of sentience (the capacity to have feelings, such as pain, love, hate, joy, and anger, hunger, and thirst) in insects were also observed ((Gibbons et al., 2022) citing (Bateson et al., 2011; Solvi et al., 2016)).

These debates persist amid uncertainty about the legal framework, which is causing confusion and lack of clarity. A regulatory gap emerges: most meat alternatives falling under the EU Novel Foods Regulation (No. 2015/2283), notably insect-based, cell-based, and certain plant-based products, are regulated without explicit reference to environmental impacts (The European Parliament, 2015). While the previous Regulation (No. 258/97) explicitly included environmental considerations, allowing Member States to temporarily restrict or suspend the marketing or use of a novel food in the event of environmental risks (Article 12), the current framework limits such references to Recitals 2 and 29, which merely highlight the objectives of “a high level of environmental protection and the improvement of its quality” and the “reduction of the environmental impact of food production” without imposing binding obligations on operators. Moreover, authorization procedures focus primarily on food safety. Yet, beyond the conditions set out in Article 7, Regulation 2015/2283 also stipulates that the Commission may consider “any other legitimate factors relevant” when assessing an application (Articles 10, 12, and 18). This limited integration of environmental dimensions reflects what Debucquet & Friant-Perrot (Debucquet and Friant-Perrot, 2016) termed as “technoscientific vision of food”, an approach that diverges from the principles of sustainability, which encompass low environmental impact, contributions to food and nutrition security, cultural acceptability, economic fairness, and accessibility (European Commission, 2020; INRAE, 2023). Social and cultural factors also influence adoption. Despite the challenges posed by social and cultural dimensions deeply rooted in meat consumption (Asamane et al., 2021; Petrescu et al., 2017), which make dietary shifts difficult, consumers mostly perceive these alternative protein sources as sustainable food options (Wang et al., 2024) in terms of climate change impact, healthy food choices, and ethical consumption practices (Grossmann and Weiss, 2021). Barnés-Calle et al. (2025) showed that reducing the product's environmental impact and improving animal welfare were among the main drivers of the consumption of meat alternatives (including insect-, plant-, and cultured meat products). Furthermore, the Sixth Assessment Report (AR6) of the Intergovernmental Panel on Climate Change (IPCC) noted that emerging food technologies could significantly reduce greenhouse gas emissions from food production, reduce land and water consumption, and address animal welfare concerns (Pathak et al., 2022). Factors such as deliberate decision-making, familiarity (Kröger et al., 2022), and emotional considerations have been recognized as influential in the adoption of meat alternatives (Onwezen et al., 2021). However, the consumption of new protein alternatives is hindered by, for example, food neophobia and dietary factors (De Koning et al., 2020; Petrescu‐Mag et al., 2022). Given the complex set of factors influencing consumers' preferences, understanding consumers' willingness to adopt these alternatives is relevant for promoting sustainable dietary transitions.

Previous research has examined a wide range of influences on consumer adoption of one or more meat alternatives. Perceptions, obstacles, and motivations for food choices, attitudes, sensory attributes, market segmentation, socio-demographic characteristics, knowledge levels, trust, purchase intention, perceived risks, or planned behavior are some of these factors (Wang et al., 2024; Begho et al., 2023; Demartini et al., 2024; Xueyun et al., 2024). As sustainability becomes a central theme in consumer markets (Nascimento and Loureiro, 2024), food labeling, including sustainability labels, is an important communication tool. Beyond their pivotal role in the industrialization of food (Cardon et al., 2019), labels can convey environmental, ethical, and production information, reducing uncertainty and guiding choices (Huang and Uehara, 2023; Van Loo et al., 2020). Labels provide assurance and transparency, addressing consumer concerns about product quality and ethical standards. Trust in labeling helps bridge the gap between consumer uncertainty and informed choices, particularly in assessing characteristics that are often complex or difficult for consumers to evaluate independently (Monaco et al., 2024). Several studies indicate that consumers choose meat alternatives not only for health reasons, but also due to environmental concerns such as climate change and animal welfare (Pakseresht et al., 2022; Sanchez-Sabate and Sabaté, 2019; Seo et al., 2023). Moreover, EU law (Article 13 TFEU) recognizes animals as sentient beings, requiring full regard for their welfare, a principle embedded in the “Farm to Fork” strategy's goal of ensuring sustainability and improved welfare throughout an animal's life. Consumers' interest in higher welfare standards, particularly at slaughter, is well documented (e.g., recital 50 of Regulation No. 1169/2011 on the provision of food information to consumers), and the 2023 Eurobarometer shows 84 % of Europeans believe farm animal welfare should be better protected in their country (European Commission, 2023).

There remains a scarcity of empirical studies that directly compare consumers' perceptions across the specific product categories aimed at meat replacement. To encourage greater acceptance of meat substitutes, it is crucial to understand how consumers perceive these alternatives relative to conventional meat in terms of environmental impact. Building on this need, the present study focuses on four burger types – insect-based (herein after called “I_burger”; it is not available in Romania), cultured meat (“CM_burger”; cultured meat is not produced or commercialized in Romania), plant-based (“P_burger”; it is available in Romania), and beef (“B_burger”; it is available in Romania). Moreover, the study compares them under two label conditions: a sustainability label (designed to highlight resource consumption, climate change, and animal welfare) and a neutral label (without sustainability claims). The general aim is to understand the influence of sustainability labels and product type on consumers' perceptions of the product's environmental impact (evaluated across three dimensions: resource consumption, climate change, and animal welfare).

The remainder of this paper is structured as follows. Section 1.2 reviews the relevant literature, formulates research questions, and outlines study objectives. Section 2 details the methodology, including study design, participants, and measures. Section 3 presents the results of the statistical analyses. Section 4 discusses the findings in relation to existing literature and outlines implications, limitations, and directions for future research. Section 5 is dedicated to the final remarks.

## 2 Sustainability labels, product type, and consumer perceptions: A review of current evidence, formulation of research questions, and study objectives

The growing concern over environmental protection, human health, work conditions, animal welfare, and other ethical aspects of food production led to the introduction of sustainability labels. Labels serve as a means to convey a food product's sustainability attributes to consumers during purchase (Jürkenbeck, 2023). Thus, sustainability labels communicate to consumers comprehensive information about specific environmental and ethical standards, social welfare, or macroeconomic benefits when making food choices (Engels et al., 2010; Grunert et al., 2014). These labels are designed to provide consumers with information about the sustainability practices involved in the production, processing, and distribution of the product. Yet, Brown et al. (Brown et al. (2020) underline that a primary difficulty in sustainability labeling for food products is the intricate nature of the sustainability concept and the food sector in which it is applied. It is the complexity of these labels that requires more in-depth investigation to ensure they effectively inform and influence consumer choices.

In a systematic review, Potter et al. (2021) found that the use of diverse sustainability labels (ecolabels) and messaging formats was associated with increased selection and purchase of more sustainable food products. Kaczorowska et al. (2019) discovered that urban consumers with positive attitudes towards sustainability are more price-sensitive and show a restrained desire to pay higher prices for sustainability-labeled products. Consumers across five European countries perceive sustainability labels as helpful and consider animal welfare among the most important attributes when buying meat and dairy products (Ammann et al., 2024). In a study dedicated to the influence of various food labels on the decision to make the transition to a meat substitute, Carlsson et al. (2022) reported several interesting findings. Thus, they demonstrated that climate and health labels on meat substitutes can significantly increase consumer preference for these products, and distinguishing meat substitutes with such labels can influence consumer behavior. Additionally, labeling meat products with information on animal care, antibiotic use, and health benefits also played an important role in consumer choices between meat and soy-based lasagna.

Consumers may have diverse reasons for preferring sustainability-labeled products. Previous studies indicated that the purchase of labeled food products is influenced not by intrinsic and extrinsic factors (Siraj et al., 2022), including ethical considerations and environmental concerns (De Canio et al., 2021). The environmental-social cues were found to be important in consumers' food quality evaluation (Petrescu et al., 2022), quality being an important driver of consumers’ buying decisions (García-Salirrosas et al., 2024). Cho (2015) considered that consumers evaluate sustainability claims more favorably if the advertisement highlights their impact.

While some studies show that sustainability labels have a positive effect on influencing consumers' perception and choices, other studies demonstrate that their overall impact is modest and varies depending on the type of label and product (Ibanez, 2016). Even if sustainability is a widely discussed topic in consumers' food choices, it often competes with factors such as taste and health (Grunert et al., 2014). As a result, people's overall interest in sustainability does not always lead them to prioritize sustainability information when selecting food products (Grunert et al., 2014).

We can infer that a limited understanding of labels could undermine the potential influence of sustainability labels on consumers' perceptions of the environmental impact of products. Moreover, the effectiveness of these labels in influencing consumers' perceptions and behaviors remains a topic of debate, varying widely due to the diverse types of information they provide and the different contexts in which they are used (Ibanez, 2016). This inconsistency and modest impact across contexts highlight the need for focused research on whether and how sustainability labels influence consumers' perception of a product's environmental impact. Therefore, the study intends to answer the following research question (RQ) about meat alternatives and meat products: *“RQ1. Does the presence of a sustainability label influence consumers' perception of the environmental impact of a product?”*

The type of product also plays a significant role in shaping consumers' perceptions of its environmental impact. In a review about the drivers of consumers’ acceptance of meat alternatives, factors such as product type and country of origin were found to influence consumer perceptions of plant-based, seaweed-based, and insect-based meat products as healthier and more sustainable options (Siddiqui et al., 2023). In a comparison between participants from Germany, France, and the United Kingdom, individuals who avoid meat or follow a flexible diet view plant-based products more favorably than those who eat meat, particularly regarding taste, texture, protein content, and environmental benefits (Michel et al., 2021); likewise, they anticipated that pea and algae burgers would be less flavorful but offer greater health benefits and be more eco-friendly than traditional beef burgers. A study on Swiss consumers revealed that respondents significantly underestimated the environmental impact of meat and overestimated that of plant-based products (Hartmann et al., 2022). Another study on Swiss consumers found that they viewed minced meat substitutes as more environmentally friendly than various meat products, yet considered organic chicken breast and organic pork strips to be more sustainable than tofu or falafel (Lazzarini et al., 2016). Erraach et al. (2021) found that consumers exhibit positive attitudes towards olive oil with sustainability labels and are willing to pay a premium for it. However, the main drivers of this behavior are often unrelated to sustainability, suggesting that product-specific factors significantly influence consumer perceptions. Consumer perceptions of plant-, seaweed-, and insect-based meat products as healthier and more sustainable options are influenced by factors such as product type and country of origin (Siddiqui et al., 2023; Lazzarini et al., 2016, 2017). In qualitative research, Petrescu-Mag et al. (2025a) reported that some participants expressed doubts about the long-term viability of technologies for meat alternatives production, raising concerns that they might fall short of sustainability goals and potentially cause unexpected environmental consequences.

These findings demonstrate that product type can greatly affect how the product is perceived. Yet, direct comparisons of multiple alternative and conventional meat products within the same framework remain scarce. Given these findings, this study seeks to answer the second research question: “*RQ2. Does the product type (I_burger, CM_burger, P_burger, and B_burger) influence consumers’ perception of the environmental impact of a product?”*

Although previous research has highlighted the role of healthiness, safety, and nutritional content in shaping consumer decisions about alternative protein sources, little is known about how sustainability labels interact with different product types to influence perceptions of environmental impact.

The interaction between product type and the presence of sustainability labels adds another layer of complexity to consumer perception. Environmental sustainability is frequently cited as a factor influencing the selection of plant-based meat alternatives. However, it appears that health considerations generally outweigh environmental concerns when consumers opt for hybrid meat/vegetable products (Lang, 2020). Gómez-Luciano et al. (2019) highlighted that consumers' willingness to purchase alternative protein sources is more influenced by perceived healthiness, safety, and nutritional content rather than environmental factors. Other studies showed that although sustainability factors matter, consumer perceptions and acceptance of insect-based foods are shaped more strongly by emotional responses, packaging images, familiar tastes and textures, contextual factors, and positive tasting experiences (Wendin and Nyberg, 2021). This suggests that sustainability labels alone may not be sufficient to significantly alter perceptions and need to be paired with other appealing product attributes to be more effective. Macdiarmid et al. (2021) found that consumers are willing to pay more for healthier meals, but are less inclined to do so for meals with a lower carbon footprint if it means reducing the meat content. This suggests that while sustainability labels may moderate the influence of product type, other factors, such as health benefits and product composition, can have a stronger impact on consumer decisions. As concluded by Giezenaar et al. (2024), the impact of health and environmental claims found on product packaging on consumers' perceptions of plant-based alternatives compared to conventional meat remains unclear and needs further exploration. Accordingly, we identify a gap regarding the interaction between sustainability labels and product type. Understanding such interactions can clarify whether labeling can shift perceptions for certain products but not others. Consequently, the third research question asks: *“RQ3. Does the label type (sustainability label and neutral label) moderate the influence of product type (I_burger, CM_burger, P_burger, and B_burger) on consumers’ perception of the environmental impact of a product?”*

To address these research questions, the study has three objectives. The first objective is to determine whether a sustainability label influences consumers' perceptions of a product's environmental impact (in terms of resource consumption, climate change, and animal welfare) compared to a neutral label. The second objective is to investigate how product type influences consumers' perceptions of a product's environmental impact. Finally, the third objective is to examine whether the perception of a product's environmental impact differs for different products when a sustainability label is present or absent (to determine whether there is an interaction effect between product type and label) (Table 1).

**Table 1:** Correspondence between research gaps, research questions, study objectives, and contributions.

Identified research gap Why is this research needed?	Research questions What are we asking?	Study objectives How do we address these RQs?	Contribution What are we contributing to the field?
Limited evidence on whether sustainability labels influence consumers' perception of environmental impact across multiple environmental dimensions.	RQ1. Does the presence of a sustainability label influence consumers' perception of the environmental impact of a product?	Objective 1: It examines whether a sustainability label affects consumers' perception of a product's environmental impact compared to a neutral label.	Provides empirical evidence on the isolated effect of sustainability labels across three key environmental dimensions, using a controlled comparison with a neutral label.
Unclear role of product type in shaping consumers' perception of environmental impact.	RQ2. Does the product type (I_burger, CM_burger, P_burger, and B_burger) influence consumers' perception of product's environmental impact?	Objective 2: It investigates how product type influences consumers' perceptions of environmental impact.	Expands understanding of how different meat alternatives and conventional meat are perceived in terms of environmental impact, including products unavailable in the local market.
Lack of research on the interaction between sustainability labels and product type in shaping environmental perceptions.	RQ3. Does the label type (sustainability label and neutral label) moderate the influence of product type (I_burger, CM_burger, P_burger, and B_burger) on consumers' perception of a product's environmental impact?	Objective 3: It determines whether the perception of a product's environmental impact differs for different products depending on the presence or absence of a sustainability label.	Offers the first integrated analysis of how label type and product type interact to influence consumer perceptions, filling a gap in cross-factor studies on meat alternatives.

In summary, the literature reveals three main gaps. First, the existing evidence on the effectiveness of sustainability labels is mixed and often context-specific, leaving uncertainty about their role in shaping perceptions of environmental impact across diverse product categories. Second, studies predominantly investigated one or two products, while the inclusion of several meat alternatives and meat products in the same study is less frequent. Third, while many studies have examined the influence of a sustainability label or product type separately, few have investigated them in direct comparison within the same experimental framework. Little is known about the potential interaction between label type and product type, and how this interplay may affect consumer evaluations. Finally, it is common to find studies on consumers' perceptions of the environmental impact of a food product, focusing on a single environmental aspect (e.g., climate change, water consumption), whereas investigations of multiple dimensions are scarce. Addressing these gaps, this paper makes three key contributions. It simultaneously examines the effects of sustainability labels and product type on consumers’ perceptions of environmental impact, considers multiple environmental dimensions (resource consumption, climate change, and animal welfare), and tests for potential interaction effects (Table 1). By doing so, the study offers a more integrated understanding of how labeling strategies can be tailored to different meat alternatives and conventional meat products, providing both theoretical insights for consumer behavior research and practical guidance for policymakers and industry stakeholders.

## 3 Methodology

An online survey was conducted to fulfill the research objective using a convenience sample of 432 Romanian consumers. The sample included 55 % women and 45 % men, with an average age of 35.38 years. Participants' educational attainment was high: 0.93 % had completed up to 8 years of schooling, 8.33 % completed 9–12 (or 13) years, 60.65 % held a university degree, 25.23 % a master's degree, and 4.86 % a doctoral degree. Overall, more than 90 % of respondents had a tertiary education, indicating that the sample was sufficiently educated to understand the sustainability-related claims and product descriptions used in the study. All participants took part voluntarily, with their anonymity guaranteed and informed consent obtained prior to their involvement. Only individuals aged 18 or older were eligible to participate. The research protocol received approval from the Ethics Committee of XYZ University [blinded for review]. The burger was selected as the test product for several reasons. First, because one of the most effective ways to improve the currently low acceptance of insects as food is to present them in familiar formats, such as burger patties or chips [ (Gmuer et al., 2016; Schouteten et al., 2016; Tan et al., 2015) cited by (Kusch and Fiebelkorn, 2019)]. Tarrega et al. (2020) also used burgers to investigate consumers' willingness to consume meat alternatives and reduce meat consumption. Thus, they are associated with known products and visually masked or hidden in foods rather than visible as parts or whole, which increases acceptance (Florenca et al., 2022). Burgers are a common and easily comparable category of minced meat products in the market. Their widespread familiarity among consumers facilitated reliable perception testing without introducing novelty-related bias. Burgers are also standardized, scalable products, making them relevant for assessing sustainability perceptions tied to mass production. This choice thus balanced experimental control with ecological validity. Second, we needed to present the same image for all product types, and minced meat was a suitable (credible) way to represent all tested products (made of insects, plants, cultured meat, and beef) using the same image. Among various minced meat products, the burger was selected for analysis based on a pre-test (N = 19 Romanian consumers; 21 % men; average age: 36.7 years). The pre-test investigated the consumption frequency of three common products made from minced meat: burgers, meatballs, and souvlaki (on a scale from 1 = Never to 7 = Very often). Means and standard deviation for the products included in the pre-test were: for burgers, M = 4.37, SD = 1.67; meatballs, M = 3.89, SD = 1.99; souvlaki, M = 2.79, SD = 1.54. We ensured the questionnaire's content validity by basing its items on well-established sustainability dimensions (resource consumption, climate change, animal welfare). To ensure face validity, the items and instructions were reviewed by six academic experts to confirm that the wording accurately reflected the intended concepts and was appropriate for a general consumer sample. The experts had a PhD or master degree and experience in consumer research – quantitative or qualitative. Their background was in economic, environmental, or agronomic sciences. Based on their feedback, several wording refinements were made to improve comprehensibility and reduce ambiguity. The experts were independent from the study and affiliated with recognized universities. Then, a pilot test was conducted to confirm the questionnaire's clarity, and necessary revisions were implemented based on the feedback (see Table 1A for questions and answer options). As our goal was to test experimental effects (product type × label), not to develop a psychometric scale, this approach was appropriate for ensuring validity.

Although two of the tested products (insect-based and cultured meat burgers) are not yet available in Romania, their inclusion was intentional to assess consumer perceptions of both familiar and unfamiliar protein sources within the same experimental framework. Reliability was ensured by using standardized visual stimuli and consistent descriptions across all product types, thus minimizing interpretative bias. Participants were clearly informed about each product's nature, allowing valid attitudinal responses even without direct consumption experience. Similar approaches (Petrescu‐Mag et al., 2022; Rolland et al., 2020; Siegrist et al., 2018; Mancini and Antonioli, 2019; Verbeke et al., 2015) are established in consumer perception research, where hypothetical or conceptually known products are used to evaluate consumers' perceptions. Reviews of consumers' perceptions and acceptance of cultured meat (Bryant and Barnett, 2018; Siddiqui et al., 2022) show that the majority of cultured-meat perception studies rely on “scenario-based, image-based, or information-based” evaluations, considered valid proxies for consumer intentions prior to market introduction. Because consumers' assessments of environmental impact depend primarily on the information provided and not on direct sensory experience, the hypothetical design provides a valid and conservative measure of perceived sustainability across product types. Therefore, the results reliably capture consumers' perceived environmental impact, aligning with the study's stated aim.

The independent variables were the product type (I_burger, CM_burger, P_burger, and B_burger) and label (sustainability label and neutral label). The dependent variables represented the product's environmental impact and included resource consumption levels, the impact of the production process on climate change, and the impact of the production process on animal welfare. A 4 (product type) × 2 (label) between-subjects design was employed, resulting in eight groups (Table 2), each with 54 responses.

**Table 2:** Images presented to respondents.

Label condition/Product type	Sustainability label	Neutral label
Insect-based burger		
Cultured meat burger		
Plant-based burger		
Beef meat burger		

Each group of participants viewed the image of one of the four burger types (each type made of one main ingredient – cultured meat, plants, insects, and beef meat), accompanied by either a sustainability label (containing the product name, health and sustainability claims, weight, and a generic expiration date) or a neutral label (containing the product name, weight, and a generic expiration date) (Table 2). People rated the perceived environmental impact (i.e., separately for resource consumption, climate change, and animal welfare) of each product on a 7-point Likert scale (e.g., “What effect do you think the production of insect burgers has on climate change if they are produced on a large scale?”; 1 = They contribute very little to climate change, …., 7 = They contribute very much to climate change) (Table 1A, Annex).

To maintain consistency across product type conditions, the authors used a real burger image, created the sustainability and neutral labels (left side of the images, Table 2), and a small label with the weight and generic expiration date (right side of the images, Table 2). The shape and size of the labels, the packaging, and the product itself were identical in all conditions to avoid confounding effects (Vermeir and Roose, 2020). In designing the sustainability label, the authors were inspired by the Planet Score label (https://www.planet-score.org/en/), which uses categories (e.g., biodiversity, climate, breeding methods) for product evaluation like those included in the sustainability label used in this study (i.e., resource consumption, climate change, animal welfare). In total, three sustainability indicators were included in the sustainability label: “Care for animals”, “Savings for energy, water, and land”, and “Stop climate change” (Table 2). An interpretative label was placed at the bottom of the sustainability label because studies demonstrated that interpretative labels (e.g., traffic light systems) are effective in guiding consumers toward better (i.e., healthier, more sustainable) food choices [ (Cecchini and Warin, 2016) cited by (Jürkenbeck, 2023)]. In the environmental context, an interpretative label interprets the environmental impact of a product by giving an evaluation or judgment, and can include (a) environmental-specific indicators (e.g., low carbon emissions; forest protected) and (b) summary indicators (Planet Score, Eco-Score, Eco Impact, or Eaternity). In contrast, an informative label provides factual information without interpretation or guidance, and the consumer must interpret the meaning themselves (e.g., 70 l of water; 150 Kcal/100 g of product).

A two-way multivariate analysis of variance (MANOVA) was conducted to examine whether the effect of product type on perceived healthiness and environmental impact differed depending on the label condition. This analysis was appropriate given the two independent variables – product type (I_burger, CM_burger, P_burger, and B_burger) and label (sustainability label and neutral label) – and three dependent variables: resource consumption, climate change associated with product production, and animal welfare (Laerd Statistics, 2017). The dependent variables were moderately correlated (values ranging from −.454 to .786), which supported the use of a two-way MANOVA instead of running separate two-way ANOVAs for each dependent variable. In this study design, the product type was the focal variable, and the label condition was the moderator. To observe whether the education level and living environment have an impact on consumers' perception of a product's environmental impact, a three-way ANOVA was run for each type of impact (considering product type, label, and education as independent variables and resource consumption/climate change/animal welfare as the dependent variable). The study used a between-subjects design. Data was analyzed using SPSS Statistics (version 24).

## 4 Results

Respondents evaluated the environmental impact of meat alternatives as lower compared to that of a beef burger (Table 3). Two-way MANOVA revealed a statistically significant interaction effect between label and product type on the combined dependent variables, F (9, 1027) = 2.230, p = .018, Wilks' Λ = .954, partial η^2^ = .016. Specifically, there was a statistically significant interaction effect between label and product type for climate change [F (3, 424) = 3.882, p = .010, partial η2 = .026], but not for resource consumption [F (3, 424) = 2.198, p = .088, partial η2 = .015] and animal welfare [F (3, 424) = 1.257, p = .289, partial η2 = .009]. Statistical significance was set at p < 0.0167 after using a Bonferroni correction. The 0.0167 limit was set by dividing the current level of statistical significance (i.e., p < .05) by the number of dependent variables being tested (i.e., three dependent variables in our case; p < .05 ÷ 3). Because we have at least one significant interaction, we will interpret the simple main effects.

**Table 3:** Consumers’ perception of the environmental impact of four products (N = 54 for each group).

Environmental impact	Product type	Label type	Mean	SD
Resource Consumption (Scale: 1 = Very small, …, 7 = Very high) Higher mean = higher negative impact	I_burger	Sustainability Label^a^	2.93	1.334
		Neutral Label^l^	2.65	1.456
	CM_burger	Sustainability Label^b^	3.20	2.293
		Neutral Label^m^	3.07	2.179
	P_burger	Sustainability Label^c^	2.61	1.123
		Neutral Label^n^	2.89	1.462
	B_burger	Sustainability Label^A,B,C^	5.35	1.507
		Neutral Label^L,M,N^	5.46	1.383
Climate change (Scale: 1 = Very small contribution to climate change, …, 7 = Very high contribution to climate change) Higher mean = higher negative impact	I_burger	Sustainability Label^[1#],d,J^	3.30	1.586
		Neutral Label (Runhaar, 2025)^,o^	2.93	1.768
	CM_burger	Sustainability Label^e^	2.72	2.032
		Neutral Label^p^	3.02	2.023
	P_burger	Sustainability Label^f,j^	2.30	1.021
		Neutral Label^q^	2.52	1.177
	B_burger	Sustainability Label^D,E,F^	5.20	1.559
		Neutral Label^O,P,Q^	5.26	1.469
Animal welfare (Scale in the questionnaire: 1 = No respect at all for animal welfare, …, 7 = Very high respect for animal welfare∗) Values are reversed for the “animal welfare” variable in this table and the rest of the analyses and data interpretation => Higher mean = higher negative impact on animal welfare in this table∗	I_burger	Sustainability Label^g,K^	4.06	1.937
		Neutral Label^r,U,V^	4.13	1.649
	CM_burger	Sustainability Label^h^	3.48	2.213
		Neutral Label^s,u^	2.87	1.812
	P_burger	Sustainability Label^i,k^	2.83	1.370
		Neutral Label^t,v^	3.07	1.372
	B_burger	Sustainability Label^G,H,I^	5.78	1.383
		Neutral Label^R,S,T^	5.61	1.795

Notes: ∗ The discussion about “animal welfare” refer to “negative impact on animal welfare”, thus, considering the reversed values.

Superscript numbers and letters indicate pairs of products between which there is a statistically significant difference; a superscript number with a # next to it indicates the product with a higher negative impact compared to its pair (marked with the same number, but without the #); a superscript capital letter indicates the product with a higher negative impact compared to its pair (marked with the same letter, but as a small letter; e.g., “A” and “a” letters signal that an I_burger with a sustainability letter has a perceived higher resources consumption compared to an I_burger with a neutral label).

1 (number) between square brackets = Simple main effects of label: significant statistical difference between the perceived environmental impact of a product with a sustainability label and one with a neutral label (square brackets were used to avoid confusion between the number “1” and the “l” and “I” letters, which have a similar shape).

a, …, v (letter) = Simple main effects of product type: significant statistical difference between the perceived environmental impact of one product type compared to other product type; this was done in two steps, first, making comparisons between pairs of products within the group of all products with a sustainability label; second, making comparisons between pairs of products within the group of all products with a neutral label.

In the next step, simple main effects were examined in turn for label and product type. First, the simple main effects for label indicated the effects of label on each product type. This means that we are concerned with the simple main effect of label (i.e., differences in evaluations between sustainability and neutral labels) for the I_burger, CM_burger, P_burger, and B_burger. Since the label is a two-group variable (i.e., sustainability label and neutral label), these simple main effects assess the difference between the sustainability and neutral labels on the three dependent variables combined for the I_burger, CM_burger, P_burger, and B_burger. We used a Bonferroni correction again to declare statistical significance at p < .0125. This was achieved by dividing the current level of statistical significance (i.e., p < .05) by the number of simple main effects (comparisons) being tested [i.e., four in our case (label 1 *vs* label 2 for product 1; …; label 1 *vs* label 2 for product 4); p < .05 ÷ 4]. Thus, we observed that there was a statistically significant difference between sustainability and neutral labels for the perceived climate change [F (1, 424) = 10.543, p = .001, partial η2 = .024] for the I_burger (with a higher impact of the product with a sustainability label), but not for the other three products. No differences exist between labels for any of the products regarding perceived resource consumption or animal welfare (Table 3). Trends in products’ perceived environmental impact across impact types and labels are visible in Fig. 1.

**Fig. 1:**
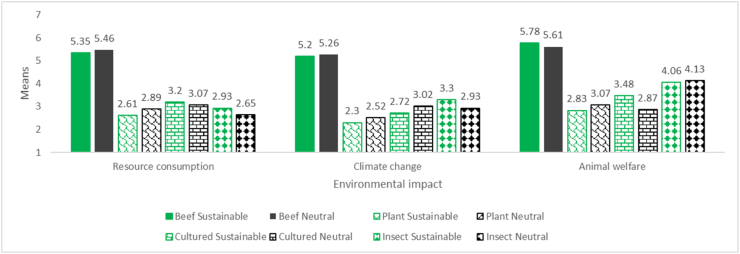
Trends in perceived environmental impact across product types and label conditions (mean values).

Second, the simple main effects for product type reveal the simple main effect of product type for each type of label (i.e., sustainability and neutral). Univariate tests showed that there was a statistically significant difference between the perceived environmental impact (for each of the three categories) for each type of label, with all p = 0.000 (Table 3; Table 4, “Sig.” column). This means that, for example, when examining products with a sustainability label, the perceived resource consumption is statistically different between at least one pair of products (e.g., I_burger vs. CM burger, or I_burger vs. P_burger, etc.). However, univariate tests do not indicate which pairs of products exhibit a difference, and post-hoc tests were conducted within the two-way MANOVA to determine which pairs differ. A Bonferroni correction was used again to declare statistical significance at p < .025. This was achieved by dividing the current level of statistical significance (i.e., p < .05) by the number of simple main effects (comparisons) being tested (i.e., two in this case because we tested differences between products 1, 2, 3, 4 for label 1 and differences between products 1, 2, 3, 4 for label 2; p < .05 ÷ 2). With all p = 0.000, all were statistically significant.

**Table 4:** Univariate tests for testing simple main effects of product type.

Dependent Variable	Label		Sum of Squares	df	Mean Square	F	Sig.	Partial Eta Squared
Resource consumption	Sustainability	Contrast	235.481	3	78.494	28.687	.000	.169
		Error	1160.167	424	2.736			
	Neutral	Contrast	302.125	3	100.708	36.805	.000	.207
		Error	1160.167	424	2.736			
Climate change	Sustainability	Contrast	266.384	3	88.795	33.423	.000	.191
		Error	1126.444	424	2.657			
	Neutral	Contrast	284.051	3	94.684	35.639	.000	.201
		Error	1126.444	424	2.657			
Animal welfare	Sustainability	Contrast	258.556	3	86.185	29.284	.000	.172
		Error	1247.870	424	2.943			
	Neutral	Contrast	254.940	3	84.980	28.874	.000	.170
		Error	1247.870	424	2.943			

Each F tests the simple effects of ProductType within each level combination of the other effects shown. These tests are based on the linearly independent pairwise comparisons among the estimated marginal means.

Pairwise comparisons were conducted twice, first for the group of products with a sustainability label, and then for those with a neutral label. The following statistical differences were observed. For the products with a sustainability label, B_burger has higher perceived resource consumption, negative contribution to climate change, and negative impact on animal welfare compared to each of the other three products (I_burger, CM_burger, and P_burger); perceived negative contribution to climate change and negative impact on animal welfare is higher for I_burger compared to P_burger (Table 3; Table 2A, Annex).

For the products with a neutral label, again, B_burger has higher perceived resource consumption, negative contribution to climate change, and negative impact on animal welfare compared to each of the other three products (I_burger, CM_burger, and P_burger); perceived negative impact on animal welfare is higher for I_burger compare to CM_burger and and P_burger (Table 3; Table 2A, Annex).

To examine whether education affects how people perceive a product's impact on resource consumption, climate change, and animal welfare, we run three-way ANOVAs for each environmental impact (with product type, label, and education as independent variables and, e.g., resource consumption as the dependent variable). First, for resource consumption, there was no statistically significant three-way interaction among label, product type, and education, F (8, 398) = .846, p = .565. This means that education does not affect (or moderate) the simple two-way interaction of label x product type on perceived resource consumption of the products. Further on, no two-way interactions (i.e., label x product type; label x education; product type x education) were found. Second, for climate change, there was no statistically significant three-way interaction, F (8, 398) = 1.139, p = .336. There was a two-way interaction between product type and education, F (10, 398) = 2.247, p = .015. Univariate tests revealed a statistically significant difference in the estimated impact of CM_burger on climate change among people with different education levels. In the next step, pairwise comparisons indicated a higher perceived impact from people with a doctoral degree compared to people with a college education, and those with 9–12 (or 13) years of education; and a higher impact from people who have studied compared to people with 9–12 (or 13) years of education. Third, regarding the perceived impact of the product on animal welfare, there was no statistically significant three-way interaction among label, product type, and education, F (8, 398) = .694, p = .697. There was a two-way interaction between product type and education, F (10, 398) = 2.138, p = .021. Univariate tests revealed a statistically significant difference in the estimated impact of CM_burger on animal welfare across education levels. In the next step, pairwise comparisons indicated a higher perceived impact from people with a doctoral degree and those with a master degree compared to people with a college education.

To observe if the living environment (rural or urban) has an impact on the way people perceive a product's impact on resource consumption, climate change, and animal welfare, we also run three-way ANOVAs. In all cases, there was no statistically significant three-way interaction among label, product type, and living environment [resource consumption: F (3, 416) = 1.298, p = .275; climate change: F (3, 416) = .829, p = .478; animal welfare: F (3, 416) = .432, p = .730]. No two-way interactions were found.”

## 5 Discussion

MANOVA results highlight several important findings regarding consumer perceptions of the environmental impact of a product with and without a sustainability label across different food product types (I_burger, CM_burger, P_burger, and B_burger), which deserve further discussion.

“RQ1. Does the presence of a sustainability label influence consumers’ perception of the environmental impact of a product?”

Our results reveal that the sustainability label has context-specific and limited influence on consumers' perception of a product's environmental impact. The sustainability labeling had a unique effect, namely on the insect burger, increasing perceived negative climate impact, but had no impact on its perceived resource consumption and animal welfare. Cultured meat, plant-based, and beef burgers elicited stable perceptions regardless of the label about the product's environmental impact (for all dimensions: resource consumption, climate change, and animal welfare).

These results indicate that sustainability communication does not operate in a vacuum but is filtered through consumers' pre-existing cognitive frames about each protein source. Rather than acting as an independent information cue, the label interacts with deeply held beliefs about what constitutes “natural”, “ethical”, or “sustainable” food. This finding provides a theoretical contribution by demonstrating that labeling effectiveness depends on the congruence between the product's perceived identity and the sustainability message it conveys. For instance, sustainability cues may trigger cognitive dissonance when attached to foods that already evoke moral ambivalence (e.g., insect-based), leading to skepticism or even reactance. The multi-dimensional nature of the sustainability label (which simultaneously emphasizes climate change mitigation, resource efficiency, and animal welfare) may have inadvertently induced a backfire effect. Prior research suggests that when a product communicates several positive attributes, consumers may experience a “too good to be true” response, questioning the credibility or realism of such claims (Huang et al., 2021). Furthermore, this paradoxical effect may reflect a form of compensatory or zero-sum belief (Huang et al., 2021). The original zero-sum belief framework posits that consumers often assume trade-offs between product attributes, such that improvements in one attribute imply sacrifices in another. While this mechanism was demonstrated in multi-label contexts (e.g., nutrition and low-carbon claims), the present findings suggest it may also operate in single-label, novel-product contexts. For novel and unfamiliar products such as insect-based burgers, perceived trade-offs may arise between sustainability claims and the product's intrinsic attributes. As a result, sustainability claims might elicit inferences of hidden trade-offs (“if it is sustainable, it must be less efficient/tasty/require other resources”).

The lack of label influence for most cases may be due to the fact that consumers often face trade-offs when products carry multiple sustainability labels, such as those for animal welfare and climate impact, because these labels may conflict, and it is uncertain how this affects their decision-making (Sonntag et al., 2023; Jürkenbeck et al., 2024; Dorisse et al., 2025). Another study highlighted that while consumers might recognize the presence of these labels, they often lack a clear understanding of their significance and implications (Grunert et al., 2014); thus, the sustainability labels played a minor role in consumers’ food choices, primarily due to a limited understanding and low usage. Further research by Cook et al. (2023) inferred that sustainability labeling can influence consumer attitudes and purchasing behaviors, although the effect sizes are generally small. They found that organic labeling, in particular, tended to lead to the highest reported willingness to pay among consumers. Furthermore, other research suggests that labeling may have unwanted consequences, such as leading to consumer distrust and dissatisfaction (Moon et al., 2017).

While other studies have shown that sustainability or eco-labels (e.g., organic) generally increase perceived environmental friendliness or healthiness across various products (Lazzarini et al., 2017; Buettner et al., 2024), including meat alternatives, our study found the opposite effect. Here, the sustainability labeling increased perceived negative impact on climate change (compared to the neutral label) and had an effect only on the I_burger. Insect-based products, relatively new to consumers, tend to elicit more malleable perceptions that are more easily influenced by sustainability labels, as consumers have fewer preconceived notions about them or less information or knowledge. Insect-based foods often suffer from food neophobia or disgust (Petrescu-Mag et al., 2025b; Abro et al., 2025), which labels alone may not overcome [e.g., claims about sustainability, as in (Naranjo-Guevara et al., 2023)]. Even when labeled as sustainable, the novelty of insect content may trigger increased perceived resource use or environmental impact, possibly as a defensive mechanism or a form of skepticism. Similarly, Li et al. (2024) showed that consumers’ perceived greenness decreased for an environmentally friendly product labeled with unfamiliar ingredients (*vs*. familiar). The absence of a label effect on how consumers evaluate animal welfare in the case of I_burger can be caused by the fact that people do not attribute strong moral standing or sentience to insects (Bastian et al., 2012), and there may be no clear mental model for insect welfare. Consequently, when people evaluate insect-based foods, animal welfare is not a salient concern, regardless of labeling.

This pattern also highlights an important psychological mechanism: when confronted with sustainability information that conflicts with affective impressions (such as disgust or moral discomfort, as suggested by previous studies for insect-based products (Hartmann and Siegrist, 2017)), consumers may rely more heavily on their emotions than on factual claims. This supports dual-process theories of judgment, where intuitive and affective pathways dominate under conditions of uncertainty or low familiarity. Thus, even accurate sustainability claims can backfire if the product evokes strong emotional resistance.

The lack of a label effect for the other three products may be explained by the fact that participants already hold stable baseline beliefs about the environmental profiles of cultured meat, plant-based, and beef burgers, and a label alone is not enough to alter their perceptions. Despite the presence of sustainability labels, consumers probably continue to rely on their intuitive judgment to evaluate the environmental impact of these foods, as previously observed by Kusch and Fiebelkorn (2019). This reliance on intuition over label information underscores the deeply ingrained nature of consumer beliefs and perceptions about these products, which are not easily swayed by external sustainability claims.

The results highlight that sustainability labels do not universally improve perceptions of alternative proteins and may even amplify negative judgments for certain products and dimensions of the environmental impact. In this case, the sustainability label possibly heightened the awareness or suspicion about the climate change effects triggered by the production of insect-based products. For insects in particular, a sustainability claim may draw attention to production realities (including large-scale animal killing) that increase perceived welfare harm. This underscores the complexity of consumer moral reasoning about food, where environmental and ethical considerations are not always aligned (Graham and Abrahamse, 2017), and the need to consider the specificity of each product and the environmental dimension being evaluated.

“*RQ2. Does the product type (*I_burger, CM_burger, P_burger, and B_burger*) influence consumers’ perception of its environmental impact?”*

Consumers view beef as having a higher environmental impact in terms of resource consumption, climate change, and animal welfare than the other three products. This perception remains consistent regardless of whether a sustainability label is present or absent. The robustness of this perception suggests that pre-existing beliefs about the impact of beef on nature are strong and not easily swayed by labeling, and play a significant role in the product's evaluation. The strong perceptions regarding beef likely stem from long-standing awareness and media coverage of the environmental impact of conventional beef production (Simmonds et al., 2024). Even when labels emphasize eco-friendly practices, the deeply ingrained perception of beef as a resource-intensive choice persists.

This result offers an important theoretical insight: product type acts as a cognitive anchor, shaping environmental evaluations even before consumers process sustainability information. Consumers’ mental models of “meat” versus “alternative proteins” serve as powerful reference frames, guiding judgments of impact more by category-based heuristics than by objective data. In the case of beef, the cultural salience of climate debates, documentary exposure, and media framing has likely consolidated a stable moral narrative that links beef consumption to environmental harm. Consequently, sustainability communication for conventional meat faces a structural credibility deficit that cannot easily be offset by labeling.

The perceptions observed in this study align with life-cycle assessment (LCA) findings, which show that beef production has higher greenhouse-gas emissions, water use, and land use than the other protein sources (Poore and Nemecek, 2018; Clark et al., 2019). Likewise, Smetana et al. (2015) demonstrated that soy meal-based and insect-based substitutes currently have the lowest environmental impact given the existing technology, and the sustainability of insect-based meat substitutes could be further enhanced with the advancement of cultivation and processing techniques. Similar to our study, other research reported that participants recognized vegetarian and insect burgers as more environmentally friendly than meat burgers (Kusch and Fiebelkorn, 2019; Smetana et al., 2015). Yet, Siegrist and Hartmann (2019) observed that the respondents might have particularly linked soy production with negative environmental impacts. Kusch and Fiebelkorn (2019) also found variation in consumers’ perceptions of the environmental impact of a fast food meal according to the type of burger patty, with meals including beef burgers receiving higher ratings than meals with vegetarian and insect burgers.

The lowest negative impact was assigned to P_burger (min 2.30, max 3.07; Table 3). Other studies also found that plant-based meat substitutes were consistently rated more favorably than other meat substitute products, but sensory and nutritional implications still exist (Szenderak et al., 2022). The lack of a statistically significant difference between P_burger and CM_burger and, in the case of resource consumption, between P_burger and I_burger may stem from the processed nature of P_burger (Giezenaar et al., 2024). Many consumers assume that minimally processed, or “natural”, foods are more environmentally sustainable than processed foods (Roman et al., 2017).

This assumption reflects a broader “naturalness bias”, wherein consumers equate low processing with ecological virtue. From a policy perspective, this bias underscores the importance of emphasizing that sustainability outcomes depend on production systems and life-cycle impacts, rather than on perceived naturalness alone. For industry, clarifying these distinctions in marketing and product labeling can help realign consumer beliefs with scientific evidence, thereby reducing misconceptions that hinder the acceptance of advanced, sustainable technologies, such as cultured or insect-based proteins.

I_burger generated interesting results. While LCAs suggest that insects can have lower climate footprints than beef (Oonincx and De Boer, 2012), the sustainability label may have heightened attention to production processes, prompting consumers to consider the scale of insect farming. In this context, regarding animal welfare, the idea that thousands of individual insects are killed per burger could inflate perceived harm, generating a “moral numeracy” effect (or quantity effect) – where people judge harm by the number of animals killed rather than sentience or suffering capacity (Wilks et al., 2021) [where “numeracy” is understood as people's capacity to comprehend and make use of quantitative information, according to (Kahan et al., 2012) citing (Lipkus et al., 2001); however, we acknowledge that the opposite also exists, namely, when people are discouraged by the big numbers or the extended effect to act, e.g. (Slovic, 2007),]. Furthermore, for climate change, in the case of the I_burger, a novel product that already triggers some uncertainty or disgust (Hartmann and Siegrist, 2017), the label may have produced a reverse halo effect (due to its novelty and disgust): even a positive sustainability cue can backfire if consumers question its credibility. This idea is supported by the statistically significant differences observed for I_burger between the two label conditions (i.e., I_burger with a sustainability label is considered here more harmful in terms of climate change compared to one with a neutral label; see Table 3).

Without a sustainability cue, judgments may be guided more by intuitive moral reasoning and gut reactions. Cultured meat, despite being technologically novel, is often understood as involving minimal or no slaughter (Pasca and Arcese, 2025), and this may have lowered its perceived animal harm in the present study. Plant-based products avoid animal use entirely, so they naturally receive the lowest harm ratings. By contrast, insect farming can cue thoughts of mass killing and “unnatural” processing, especially if consumers picture insects being harvested in large numbers. For some, this perception may overshadow the lower sentience argument, leading to higher animal harm scores even in the absence of sustainability framing.

From a marketing perspective, these findings suggest that promotional strategies for insect-based foods must carefully consider the animal-welfare dimension. While environmental benefits are present in LCAs, consumer acceptance may depend on reducing perceptions of severe harm to animal welfare. For example, reduced sentience, humane rearing methods, or the ecological benefits of insect farming can be emphasized. Future research should examine whether alternative label framings, such as biodiversity protection or efficiency claims, can improve perceptions without triggering welfare-related moral concerns.

“RQ3. Does the presence of a sustainability label moderate the influence of product type on consumers’ perception of the environmental impact?”

The results indicated a statistically significant interaction effect between label and product type on the combined dependent variables. In particular, a statistically significant interaction effect was found between label and product type for climate change, but not for the other two dependent variables. This pattern suggests that sustainability labeling influenced climate-related perceptions selectively, rather than uniformly across all sustainability dimensions. In other words, labeling altered how products were evaluated across categories, rather than shifting an overall “label effect” or an overall “product” effect in isolation. However, this interaction effect was present only for climate change. One plausible explanation is that climate change is the dimension most directly and explicitly linked in public discourse to “sustainability” messaging (Graham and Abrahamse, 2017). When participants encountered a sustainability label, they may have selectively adjusted their climate-impact ratings because the label aligned with a salient mental association. In contrast, perceptions of resource consumption and animal welfare are less likely to be automatically activated or may be shaped by more entrenched product stereotypes.

This selective sensitivity can also be interpreted through the lens of salience and cognitive accessibility: climate change dominates sustainability narratives in media and policy discourse, making it a cognitively “available” cue that consumers readily connect with environmental claims. As a result, when encountering a sustainability label, participants interpret it primarily through a climate-related frame rather than a multidimensional sustainability framework. This cognitive narrowing helps explain why labels triggered perceptual change only for climate impact, leaving resource and welfare dimensions unaffected.

The absence of interaction effects for resource consumption and animal welfare implies that these judgments were relatively stable across labeling conditions, possibly reflecting strong pre-existing beliefs. For example, beef is widely recognized as resource-intensive and harmful to animal welfare (Poore and Nemecek, 2018), and such associations may be resistant to label-based reframing. Similarly, plant-based products are strongly associated with a low animal-welfare impact, a perception unlikely to change based on sustainability claims.

Beyond labeling and product type, our results indicate a limited effect of education and living environment on a product's perceived environmental impact. Education level significantly shapes how consumers perceive the environmental impact of only one product – cultured meat. For CM_burger, while no moderating effect of education on resource consumption was found, higher-educated participants perceived cultured meat as having a greater negative impact on both climate change and animal welfare than less-educated respondents. This suggests that individuals with more advanced education may be more informed about or sensitive to the sustainability debates surrounding emerging protein technologies. By contrast, participants' living environment (urban or rural) did not significantly affect their environmental evaluations. These findings complement previous research showing that education and familiarity with sustainability concepts influence acceptance of alternative proteins. For example, Tolve et al. (2025) found that Italian consumers with higher levels of education were more open to entomophagy and more engaged with environmental issues. Our results extend this perspective to Romanian consumers, suggesting that while education enhances awareness and critical assessment of new protein sources, it may also heighten skepticism regarding their true environmental benefits.

From a marketing and policy standpoint, this finding highlights that sustainability labels may be most effective in shaping perceptions of the selected environmental dimensions, specifically climate change, rather than of dimensions that can be evaluated using other cues or heuristics. In this study, perceptions of resource consumption and animal welfare did not shift with labeling, suggesting that these judgments are more influenced by stable product-specific associations than by general sustainability messaging.

A general overview of the study's practical implications is outlined below.

i) For *policymakers*, the results suggest the need for clearer, dimension-specific sustainability labeling standards (e.g., separating climate, resource, and animal welfare impacts) to reduce misinterpretation and build public trust. They may also consider public education campaigns to align consumer perceptions with scientific evidence from life-cycle assessments. Beyond regulatory refinement, policymakers should prioritize harmonization of labeling frameworks across markets to ensure consistency and comparability. This would prevent “label fatigue” and strengthen the credibility of sustainability communication. Integrating behavioral insights, such as color coding, standardized icons, or traffic light schemes, could further enhance label comprehension among non-expert audiences. In parallel, investment in awareness programs that link sustainability choices with tangible climate outcomes (e.g., CO_2_ reduction equivalents) could help transform abstract environmental goals into everyday decision cues.
ii) For *businesses*, particularly those producing and selling novel proteins, sustainability claims should be tested and tailored to the level of familiarity with the product. Insect-based products may require supplementary messaging that addresses animal welfare concerns and explains production efficiencies. At the same time, beef producers could focus on verified improvements in environmental practices to challenge entrenched negative perceptions. For plant-based and cultured meat products, reinforcing established environmental benefits while addressing concerns about processing could strengthen acceptance. Firms should also adopt transparency-based marketing strategies that emphasize traceability, independent certification, and evidence-based storytelling, rather than relying on generic sustainability slogans. Co-designing communication campaigns with behavioral scientists could enhance message credibility and reduce consumer skepticism. Furthermore, developing multi-sensory or experiential marketing formats, such as tastings or virtual farm tours, can help bridge the gap between perceptions of sustainability and actual product experiences, thereby fostering long-term behavioral change.
iii) For *consumers*, this study highlights the importance of critically evaluating sustainability information and seeking multiple sources before forming judgments. Increased awareness of how labels are designed and what they measure can help consumers make more informed and balanced food choices. Encouraging reflective rather than intuitive decision-making, through educational initiatives, digital tools, or interactive labeling apps, could help consumers integrate sustainability cues with nutritional, ethical, and cultural considerations. In the long run, improving sustainability literacy is crucial for empowering consumers not only as buyers but also as active contributors to systemic food transformation.

### 5.1 Limitations and future research directions

Several limitations should be considered when interpreting the findings. First, the study measured perceived rather than objectively assessed sustainability outcomes; participants’ ratings may therefore reflect subjective beliefs or heuristics that do not necessarily align with life-cycle assessment data. Also, the study relied on self-reported measures, which are susceptible to social desirability bias, particularly when addressing sustainability-related topics. Third, the convenience nature of the sample and its composition may limit generalizability, as cultural background, dietary habits, and prior exposure to novel proteins can shape sustainability perceptions. Finally, the sustainability label used in this study had a specific design and wording; different label formats or messages may produce different effects.

Future research could extend these findings in several ways. Experimental studies could test a wider variety of label designs and framings, including messages emphasizing biodiversity, efficiency, or ethical production, to identify which approaches most effectively shift perceptions across different product types. Cross-cultural investigations would help determine whether the label–product interaction observed here is consistent in regions with different dietary norms and sustainability priorities. In addition, incorporating objective sustainability metrics alongside consumer perceptions could indicate where public beliefs align (or diverge) from scientific evidence. Future research could further explore how occupation or exposure to sustainability information interact with labeling and product type to shape consumer perceptions. Finally, longitudinal studies could investigate whether exposure to tailored sustainability labels influences not only immediate perceptions but also longer-term attitudes and purchasing behaviors toward novel proteins.

## 6 Conclusion

Encouraging consumers to choose sustainable meat alternatives, such as insect-based, cultured, or plant-based burgers, can be supported by carefully selecting labels that clearly indicate the product type.

The study revealed a limited and unexpected effect of the sustainability label. Consumers consider that an I_burger has a higher negative impact on climate change when it is labeled as sustainable compared to one with a neutral label, but I_burger's resource consumption and negative effects on animal welfare are similar regardless of the label. Furthermore, consumers perceive statistically equal impacts on climate change, resource consumption, and adverse effects on animal welfare of a CM_burger, P_Burger, and B_burger, respectively, when they have a sustainability label compared to the same product with a neutral label. This addresses RQ1 by indicating that the isolated effect of sustainability labels is limited and varies by product type. Thus, labels can enhance awareness and potentially improve the perceived sustainability of certain products, but they can also inadvertently heighten concerns, particularly for less familiar products like I_burger.

The comparisons across product types (in turn, for the sustainability-labeled products, and for products with a neutral label) showed that B_burger was consistently perceived as having higher resource consumption, climate change impact, and animal welfare harm compared to the meat alternative products, answering RQ2 by showing significant differences across product types regardless of labeling. This suggests a need for improved communication and education on sustainable beef production practices.

An interaction effect between label and product type was identified only for climate change, but not for resource consumption and animal welfare. These findings respond to RQ3 by demonstrating that the moderating role of label type is dimension-specific and most pronounced for less familiar products.

Overall, the study contributes to the literature by providing empirical evidence on the isolated and combined effects of sustainability labels and product types, examining multiple environmental dimensions simultaneously, and offering the first integrated analysis of label × product type interactions for meat and meat alternative products. These insights underscore the importance of examining consumers’ perceptions of specific products and labels. Such understandings are essential for designing effective education and transparent communication strategies that convey the sustainability benefits of different foods and address deeply rooted beliefs. This study further underscores the importance of targeted communication strategies that can promote the acceptance of alternative protein sources and facilitate the transition toward a more sustainable food future.

## Consent to participate

Survey participants gave their informed consent to participate.

## Consent to publish

All authors agree with the publication of the present paper. No other publishing consents are applicable.

## Ethical statement

The research received Ethical approval no 7151/May 24, 2024 from Babes-Bolyai University, Cluj-Napoca, Romania.

## Funding. Acknowledgment

This work was supported by a grant from the Romanian Ministry of Research, Innovation and Digitization, CNCS/CCCDI-UEFISCDI, project number ERANET-JPI-HDHL-ComMEATted, within PNCDI IV.

## CRediT

Dacinia Crina Petrescu: Conceptualization, Methodology, Data collection, Formal analysis, Data curation, Visualization, Writing – original draft, Writing – review & editing; Ruxandra Malina Petrescu-Mag: Conceptualization, Methodology, Project administration, Funding acquisition, Writing – original draft, Writing – review & editing; Melis Aras: Writing – review & editing; Marius Bota: Writing – review & editing; Alexandru Sut: Data collection.

## Declaration of competing interest

The authors declare that they have no known competing financial interests or personal relationships that could have appeared to influence the work reported in this paper.

## Figures and Tables

**Table 1A:** Questions used in this study

Question	Answer options
What do you think is the consumption of resources (water, energy, etc.) associated with the production of insect/cultured meat/plant-based/beef burgers^a^ if they are produced on a large scale?	1 = Very low resources consumption, …, 7 = Very high resources consumption
What effect do you think the production of insect/cultured meat/plant-based/beef burgers^a^ has on climate change if they are produced on a large scale?	1 = They contribute very little to climate change, …., 7 = They contribute very much to climate change
What is the effect of the insect/cultured meat/plant-based/beef burgers^a^ production on animal welfare if they are produced on a large scale?	1 = They do not respect at all the animal welfare, …, 7 = They respect very much the animal welfare (these answers were reversed in the analyses; results and discussion rely on reversed data)
Gender	Feminine; Masculine; Other/I prefer not to answer
Age	……… (open answer)
Education	1 = max 8 years of education; 2 = between 9 and 12 (or 13) years of education; 3 = college; 4 = master; 5 = doctoral studies
Living environment	1 = Urban; 2 = Rural

The following information was provided to the respondents who saw an insect burger:

The following image shows an insect burger. Some insects can be used as human food. There are countries where people consume them regularly. In the European Union, there are four species of insects approved for human consumption [*Tenebrio molitor* (larva of the yellow mealworm), *Locusta migratoria* (migratory grasshopper), *Acheta domesticus* (house crickets), and *Alphitobius diaperinus* (lesser mealworm); in frozen, dried, and powdered form].

The following information was provided to the respondents who saw a cultured meat burger:

“Cultured meat (also called artificial meat, lab-grown meat, synthetic meat, in vitro meat) aims to mimic conventionally produced meat. It is obtained by culturing (stem) cells and tissue. Specifically, artificial meat is produced from a few animal cells. They are multiplied in a bioreactor using a growth medium. Therefore, artificial meat is produced in a totally different way than conventional methods of animal husbandry."

a Each group of respondents saw only one product name (insect burgers, cultured meat, plant-based burgers, or beef burgers). The same product name and its corresponding image (see Table 2) were used consistently across all three questions for that group.

**Table 2A:** Pairwise Comparisons for the differences between pairs of product types for each dependent variable (environmental dimension) and for each type of label

Dependent Variable	Label	(I) ProductType	(J) ProductType	Mean Difference (I-J)	Std. Error	Sig.^b^	95 % Confidence Interval for Difference^b^	
							Lower Bound	Upper Bound
Resource consumption	Sustainability	I_burger	2	2.776E-16	.318	1.000	−.844	.844
			3	.593	.318	.380	−.251	1.436
			4	−2.148∗	.318	.000	−2.992	−1.304
		CM_burger	1	−2.776E-16	.318	1.000	−.844	.844
			3	.593	.318	.380	−.251	1.436
			4	−2.148∗	.318	.000	−2.992	−1.304
		P_burger	1	−.593	.318	.380	−1.436	.251
			2	−.593	.318	.380	−1.436	.251
			4	−2.741∗	.318	.000	−3.585	−1.897
		B_burger	1	2.148∗	.318	.000	1.304	2.992
			2	2.148∗	.318	.000	1.304	2.992
			3	2.741∗	.318	.000	1.897	3.585
	Neutral	I_burger	2	−.667	.318	.221	−1.510	.177
			3	−.481	.318	.787	−1.325	.362
			4	−3.056∗	.318	.000	−3.899	−2.212
		CM_burger	1	.667	.318	.221	−.177	1.510
			3	.185	.318	1.000	−.659	1.029
			4	−2.389∗	.318	.000	−3.233	−1.545
		P_burger	1	.481	.318	.787	−.362	1.325
			2	−.185	.318	1.000	−1.029	.659
			4	−2.574∗	.318	.000	−3.418	−1.730
		B_burger	1	3.056∗	.318	.000	2.212	3.899
			2	2.389∗	.318	.000	1.545	3.233
			3	2.574∗	.318	.000	1.730	3.418
Climate change	Sustainability	I_burger	2	.741	.314	.112	−.091	1.572
			3	1.167∗	.314	.001	.335	1.998
			4	−1.741∗	.314	.000	−2.572	−.909
		CM_burger	1	−.741	.314	.112	−1.572	.091
			3	.426	.314	1.000	−.406	1.257
			4	−2.481∗	.314	.000	−3.313	−1.650
		P_burger	1	−1.167∗	.314	.001	−1.998	−.335
			2	−.426	.314	1.000	−1.257	.406
			4	−2.907∗	.314	.000	−3.739	−2.076
		B_burger	1	1.741∗	.314	.000	.909	2.572
			2	2.481∗	.314	.000	1.650	3.313
			3	2.907∗	.314	.000	2.076	3.739
	Neutral	I_burger	2	−.574	.314	.408	−1.406	.257
			3	−.074	.314	1.000	−.906	.757
			4	−2.815∗	.314	.000	−3.646	−1.983
		CM_burger	1	.574	.314	.408	−.257	1.406
			3	.500	.314	.670	−.331	1.331
			4	−2.241∗	.314	.000	−3.072	−1.409
		P_burger	1	.074	.314	1.000	−.757	.906
			2	−.500	.314	.670	−1.331	.331
			4	−2.741∗	.314	.000	−3.572	−1.909
		B_burger	1	2.815∗	.314	.000	1.983	3.646
			2	2.241∗	.314	.000	1.409	3.072
			3	2.741∗	.314	.000	1.909	3.572
Animal welfare (negative impact)	Sustainability	I_burger	2	.574	.330	.497	−.301	1.449
			3	1.222∗	.330	.001	.347	2.097
			4	−1.722∗	.330	.000	−2.597	−.847
		CM_burger	1	−.574	.330	.497	−1.449	.301
			3	.648	.330	.302	−.227	1.523
			4	−2.296∗	.330	.000	−3.171	−1.421
		P_burger	1	−1.222∗	.330	.001	−2.097	−.347
			2	−.648	.330	.302	−1.523	.227
			4	−2.944∗	.330	.000	−3.820	−2.069
		B_burger	1	1.722∗	.330	.000	.847	2.597
			2	2.296∗	.330	.000	1.421	3.171
			3	2.944∗	.330	.000	2.069	3.820
	Neutral	I_burger	2	1.259∗	.330	.001	.384	2.134
			3	1.056∗	.330	.009	.180	1.931
			4	−1.481∗	.330	.000	−2.357	−.606
		CM_burger	1	−1.259∗	.330	.001	−2.134	−.384
			3	−.204	.330	1.000	−1.079	.671
			4	−2.741∗	.330	.000	−3.616	−1.866
		P_burger	1	−1.056∗	.330	.009	−1.931	−.180
			2	.204	.330	1.000	−.671	1.079
			4	−2.537∗	.330	.000	−3.412	−1.662
		B_burger	1	1.481∗	.330	.000	.606	2.357
			2	2.741∗	.330	.000	1.866	3.616
			3	2.537∗	.330	.000	1.662	3.412

Based on estimated marginal means.

∗. The mean difference is significant at the .05 level.

b. Adjustment for multiple comparisons: Bonferroni.

**figs1:**
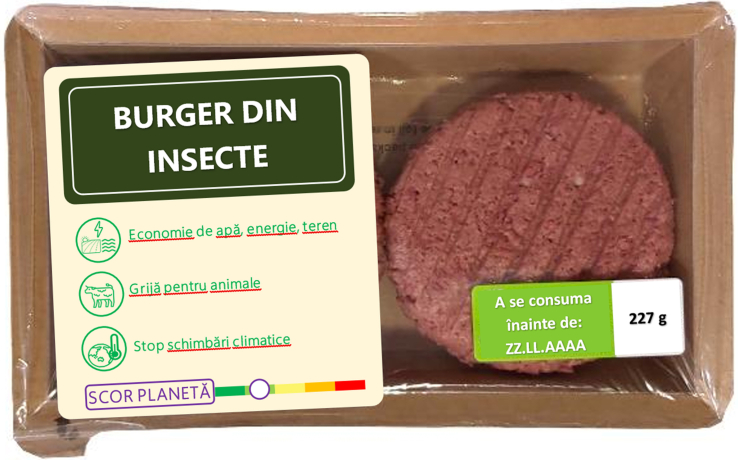


**figs2:**
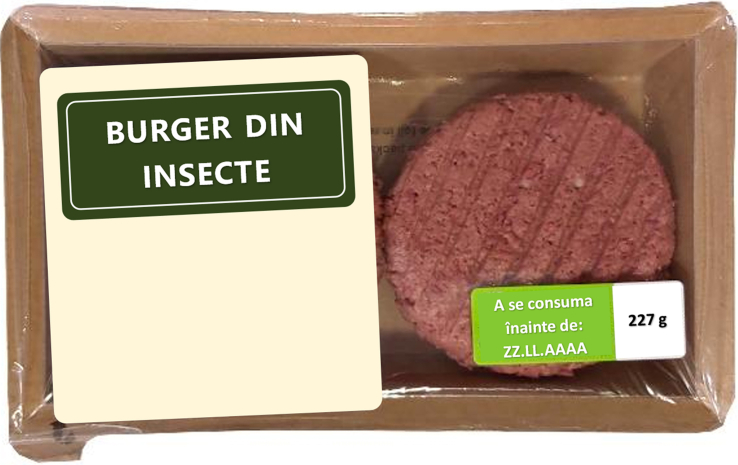

